# History of Envenoming Therapy and Current Perspectives

**DOI:** 10.3389/fimmu.2019.01598

**Published:** 2019-07-10

**Authors:** Manuela B. Pucca, Felipe A. Cerni, Rahel Janke, Erick Bermúdez-Méndez, Line Ledsgaard, José E. Barbosa, Andreas H. Laustsen

**Affiliations:** ^1^Medical School, Federal University of Roraima, Boa Vista, Brazil; ^2^Department of Biotechnology and Biomedicine, Technical University of Denmark, Kongens Lyngby, Denmark; ^3^Department of Biochemistry and Immunology, Medical School of Ribeirão Preto, University of São Paulo, Ribeirão Preto, Brazil; ^4^Facultad de Farmacia, Universidad de Costa Rica, San José, Costa Rica

**Keywords:** envenoming therapy, antivenom, antibodies, antiserum, hybridoma technology, phage display, recombinant antivenom, antivenom history

## Abstract

Each year, millions of humans fall victim to animal envenomings, which may either be deadly or cause permanent disability to the effected individuals. The Nobel Prize-winning discovery of serum therapy for the treatment of bacterial infections (tetanus and diphtheria) paved the way for the introduction of antivenom therapies for envenomings caused by venomous animals. These antivenoms are based on polyclonal antibodies derived from the plasma of hyperimmunized animals and remain the only specific treatment against animal envenomings. Following the initial development of serum therapy for snakebite envenoming by French scientists in 1894, other countries with high incidences of animal envenomings, including Brazil, Australia, South Africa, Costa Rica, and Mexico, started taking up antivenom production against local venomous animals over the course of the twentieth century. These undertakings revolutionized envenoming therapy and have saved innumerous patients worldwide during the last 100 years. This review describes in detail the above-mentioned historical events surrounding the discovery and the application of serum therapy for envenomings, as well as it provides an overview of important developments and scientific breakthroughs that were of importance for antibody-based therapies in general. This begins with discoveries concerning the characterization of antibodies, including the events leading up to the elucidation of the antibody structure. These discoveries further paved the way for other milestones in antibody-based therapies, such as the introduction of hybridoma technology in 1975. Hybridoma technology enabled the expression and isolation of monoclonal antibodies, which in turn formed the basis for the development of phage display technology and transgenic mice, which can be harnessed to directly obtain fully human monoclonal antibodies. These developments were driven by the ultimate goal of producing potent neutralizing monoclonal antibodies with optimal pharmacokinetic properties and low immunogenicity. This review then provides an outline of the most recent achievements in antivenom research, which include the application of new biotechnologies, the development of the first human monoclonal antibodies that can neutralize animal toxins, and efforts toward creating fully recombinant antivenoms. Lastly, future perspectives in the field of envenoming therapies are discussed, including rational engineering of antibody cross-reactivity and the use of oligoclonal antibody mixtures.

## Introduction

**Figure 1:**
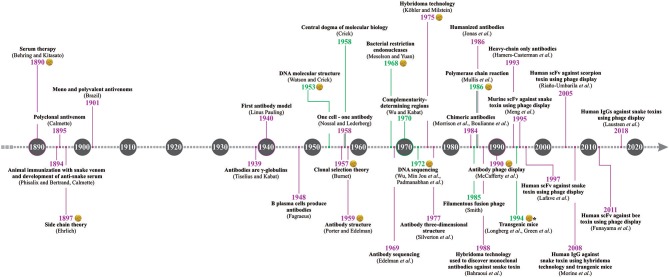
Antivenom history timeline. The timeline presents the most important discoveries related to antivenom research and development, including groundbreaking discoveries within immunology (years in purple) and molecular biology (years in green). The discoveries awarded with the Nobel Prize are indicated with the Alfred Nobel medal, but does not mean that all the researchers involved were laureated with the prize. *Gene modification discovery in mice was awarded with the Nobel Prize, and not the generation of fully human antibodies in transgenic mice.

The discovery of serum therapy has paved the way for many human therapies, including envenoming therapy. Thanks to ingenious experimentation performed more than 120 years ago, several antivenoms are available for treating accidents caused by venomous animals (1). Current antivenoms are heterologous and polyclonal in nature, as they are still manufactured via hyperimmunization of large domesticated animals, such as horses, sheep, donkeys, or camels (2). However, over more than one century of antivenom production, many important scientific discoveries and development of new technology have helped improve antivenom manufacture, as well as brought opportunities for developing fundamentally novel antivenom products (e.g., the discovery of antibody and DNA structures, hybridoma technology, polymerase chain reaction (PCR), recombinant expression of antibodies, and phage display technology). Therefore, several researchers worldwide are currently working towards improving the manufacture and quality of both plasma-derived and non-plasma-derived antivenoms. This review presents a historical perspective of the main discoveries (Figure 1) that have impacted or may impact the development and manufacture of antivenoms.

## The Origin of Serum Therapy

**Figure 2:**
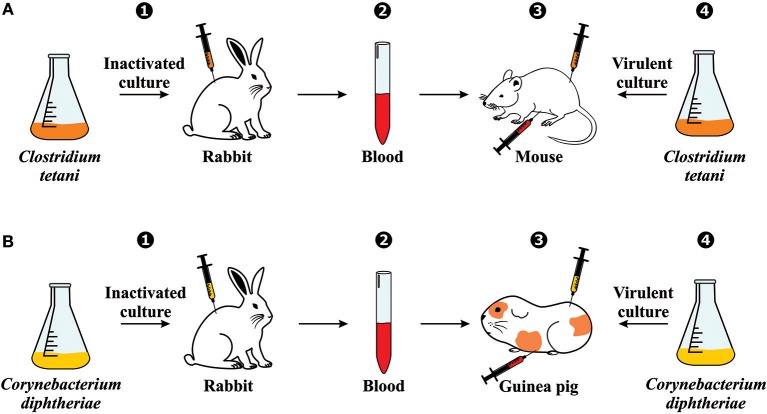
Serum therapy discovery: Experimental generation of immunity against **(A)** tetanus and **(B)** diphtheria. The experiments involved (1) immunizing rabbits against an inactivated bacteria culture; (2) collecting the blood from the immunized rabbits; (3) injecting the blood (before coagulation) into the abdominal cavity of another animal (mouse or guinea pig); (4) and inoculating the virulent culture in the same group of animals.

The use of serum therapy began in 1890 when Emil von Behring and Shibasaburo Kitasato published their groundbreaking paper on tetanus immunity (3). Briefly summarized, their experiments consisted of (1) immunizing rabbits against an inactivated culture containing *Clostridium tetani*; (2) collecting the blood from these animals; (3) injecting the blood (before coagulation) into the abdominal cavity of mice; (4) and inoculating *C. tetani* virulent culture in the same group of mice (Figure 2A). With their findings, Behring and Kitasato put forward the theory of humoral immunity, proposing that a mediator in the blood could neutralize a foreign antigen, which was mentioned in their paper as “*Blut ist ein ganz besonderer Saft*” or “Blood is a very special juice.” One week later, Behring alone published another article on diphtheria immunity using the same design methodology. However, since mice and rats are naturally immune to diphtheria, he used guinea pigs to test the mechanism (Figure 2B) (4).

After some years, diphtheria and tetanus therapies were brought into the clinic and tested on human subjects. The first human trial with serum therapy against diphtheria was performed in 1892. However, the test only achieved limited success due to insufficient antiserum quality. In 1894, after standardization of immunization techniques with larger animals had been implemented, results from another trial involving 220 children suffering from diphtheria revealed an overall 77% cure rate when the diphtheria antiserum was used. Upon this success, Behring was soon venerated as the “savior of children” (5). In comparison, serum therapy against tetanus was only introduced to the clinic after the outbreak of World War I (1914), in which broad-scale anti-tetanic serum was administered to wounded soldiers admitted to military hospitals (6).

For his serum therapy discoveries, Behring was the first scientist to be laureated with the Nobel Prize in physiology and medicine (1895), which today is the most prestigious international symbol of scientific excellence (7). Unfortunately, Kitasato did not receive the same recognition (8).

## Important Discoveries Within Antibody Generation and Characterization

**Figure 3:**
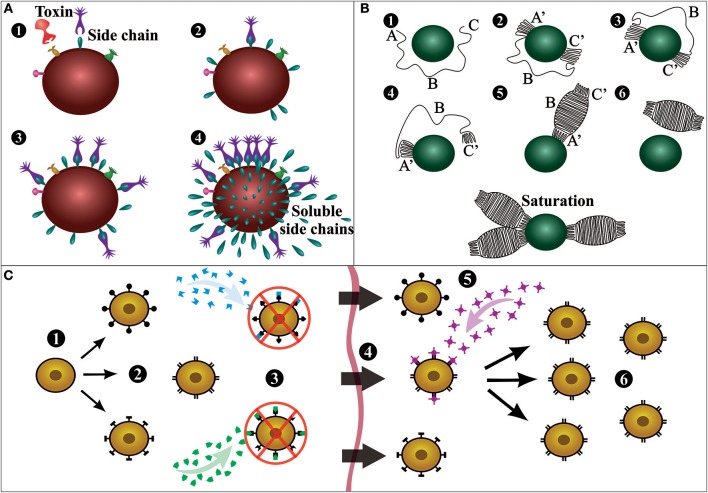
Schematic representation of important antibody discovery theories. **(A)** Ehrlich's side chain theory: Immune cells present a vast array of receptors or side chains (1); when a toxin interacts with a specific side chain (2), the immune cell is activated and thereafter produces more side chains (3); then, the receptors are released into the bloodstream as soluble side chains or magic bullets (4). **(B)** Linus Pauling's instructional model: Six different postulated stages of antibody formation as the result of interaction with an antigen. An antigen is held in place at the site of antibody production, and the antibody is generated around the antigen molecule (1). The ends of the antibody coil into a configuration complementary to groups on the antigen and attach to these complementary groups (2). The center of the chain is freed from the site of synthesis, causing one of two things to happen. If the forces between the ends of the chain are sufficiently strong, both ends will continue to be attached to the antigen, and the antibody will never be completed (3). If the forces between the ends of the chain and the antigen are weak, one end will dissociate from the antigen (4). Assuming one end of the chain dissociates from the antigen, the center of the chain coils into its most stable configuration, making a complete antibody (5). Eventually, the antibody will dissociate from the antigen and float away (6). There is also shown (lower) an antigen molecule surrounded by attached antibody molecules (saturation). **(C)** Burnet's clonal selection theory: A hematopoietic stem cell (1) undergoes differentiation and genetic rearrangement to produce immature lymphocytes with many different antigen receptors (2); the cells that bind to antigens from the body's own tissues (self-antigens) are destroyed (3), while the rest mature into inactive lymphocytes (4); cells that are activated by a foreign antigen (5) can produce many clones of themselves (6). This figure was based on the original diagrams prepared by the respective scientist.

Although Behring and Kitasato were the pioneers of serum therapy, Paul Ehrlich was the scientist responsible for the first large-scale production of antiserum. Ehrlich established that the use of low but increasing quantities of a toxin rendered animals immune against further lethal doses of the same toxin, formulating the concepts of active and passive immunization, of which the mechanisms were explained by his side chain theory (Figure 3A). The concept of the side chain theory is that antibodies are produced by white blood cells as side chains (i.e., receptors) on the cell membrane, and when getting into contact with a toxin (i.e., antigen) these side chains should be released to the blood stream as magic bullets (i.e., antibodies). Moreover, he hypothesized that cell-bound receptors would induce the cell to produce more receptors with the same specificity. Ehrlich published the first part of his side-chain theory in 1897, but the theory was only recognized later (1900) when he gave a lecture to the Royal Society in London (9–14).

In 1939, Arne Tiselius and Elvin Kabat demonstrated that antibodies are γ-globulins using electrophoresis (15), and in the following year, Linus Pauling proposed the first antibody instructional model (Figure 3B). Pauling assumed that antibodies contain the same polypeptide chains as normal globulins, and that they differ only in the way that the chain is coiled in the molecule (16). In 1948, in her doctoral thesis, Astrid Fagraeus described that plasma B cells are responsible for antibody generation (17). Her ideas were further developed in 1957 with Frank Burnet's clonal selection theory (Figure 3C). Burnet proposed that antibody-producing cells make antibodies of only one specificity, which is determined before it encounters the antigen. However, when these cells encounter a specific antigen, they can divide by clonal proliferation and thus selectively increase antibody abundance (i.e., clonal selection) (14, 15). Gustav Nossal and Joshua Lederberg soon after confirmed that individual plasma cells only produce antibodies with specificity against one antigen (18).

The first molecular structure of antibodies was described by Gerald Edelman and Rodney Porter in 1959. Porter's experiments consisted of using the enzyme papain to cleave a rabbit γ-globulin (immunoglobulin G or IgG) into three pieces of about 50,000 Da each, corresponding to the two Fab (antigen-binding) and constant Fc (crystallizable) fragments. Moreover, he observed that the crystals of the Fc fragments coming from antibodies with different specificities were practically homogeneous, and that the lack of capacity of Fab fragments to form crystals resulted from their structural heterogeneity and differences in their amino acid sequences (19). Moreover, Edelman's experiments consisted of reducing the disulfide bonds of antibodies in the presence of denaturing agents, resulting in the dissociation of the molecule into smaller pieces, now known to be the light (L) and heavy (H) chains (20). Ten years later (1969), Edelman was also the key person responsible for the elucidation of the complete amino acid sequence of a human γG1 immunoglobulin (21), which was validated in 1977 with the three-dimensional structure of an antibody determined by Silverton et al. (22). In 1970, Tai Wu and Elvin Kabat explored the variable regions of immunoglobulin chains, resulting in the discovery of the complementarity-determining regions (CDRs) (23). Other important discoveries were made between the 1960s and 1970s, including the elucidation of the role of the thymus (24), antibody class switching (25), and the development of the enzyme-linked immunosorbent assay (ELISA) (26). However, a detailed account on antibody history can be found elsewhere (27, 28).

## The History of Antivenom Therapies

The history of antivenom begins with the work of the French physician Albert Calmette in the late Nineteenth century. Calmette was given the opportunity to direct a new Pasteur Institute in Saigon, French Indochina (now Ho Chi Minh City, Vietnam), where he started his research on vaccination against rabies and smallpox in 1891 (29). However, after the deadly invasion of several venomous cobras in a local village, Calmette was presented with the opportunity to study the venom of snakes. In the article containing his findings on the physiology of envenomation, physicochemical properties of the venom, and the effect of different chemicals on the venom, he also wrote about his first but unsuccessful attempts to induce immunity against snake venom in animals. It was not until his return to France two years later that he successfully immunized rabbits with cobra venom (29, 30). It has been argued that the preceding work of Henry Sewall on inoculation of pigeons with rattlesnake venom (31) was a contributor to Calmette's eventual success (32). Calmette described several immunization strategies, including the repeated administration of increasing doses of venom and the inoculation of mixtures of venom and sodium or calcium hypochlorite, which all resulted in the animals developing resistance to doses well above the original lethal dose of venom within weeks (33). Furthermore, he described the properties of the serum of these immunized animals as being antitoxic *in vitro*, as well as being preventive when administered to rabbits before, and being therapeutic when administered after injection of venom *in vivo*. At this point, he already suggested that his antiserum could be used to treat snakebite incidents in humans in the future. Calmette claimed that his antivenom was polyspecific based on the observations that the antiserum he raised against one snake also appeared to be effective against the venom of other species (33, 34). This was to be controverted later on by different scientists, one of the first ones being Charles Martin, who showed the inefficacy of Calmette's serum against venom from Australian snake species (35). Martin partly traced their contradicting results on antiserum specificity back to the fact that they had different understandings of the mode of action of the antiserum. While he believed the interaction between the venom and its antivenom to be direct (today known to be the antibody-target binding interaction), Calmette thought that the administration of serum indirectly leads to protection by inducing a reaction in cells of the body (36). It was only a few years later that Vital Brazil proved that snake antivenoms were species specific (37).

When Calmette first presented his results in an academic session in February of 1894 (38), two other scientists, Césaire Phisalix and Gabriel Bertrand, reported their own success in immunizing guinea pigs with viper venom and likewise described the antitoxic properties of their serum (39). Their approach, however, included inoculation with venom that had been heat-treated at temperatures between 75 and 90°C, which rendered the animals resistant to lethal doses of the same venom after just 2 days (34). Phisalix and Bertrand could, however, only achieve *in vitro* neutralization with their serum, which was due to the IgM-nature of the primary immune response they induced with their approach, whereas the serum produced with Calmette's immunization protocol over several weeks resulted in IgG format antibodies that were able to protect *in vivo* (29).

In spite of their simultaneous publication of the principle of immunization with snake venom and use of their serum as an antivenom, only Calmette receives recognition for this discovery nowadays. By 1895, Calmette had produced anti-cobra serum in donkeys in larger quantities and with higher therapeutic activity (40), and in the same year, a horse anti-cobra serum was used to successfully treat a human envenoming case for the first time (41).

The importance of Calmette's work was also realized by the Brazilian doctor Vital Brazil Mineiro da Campanha who had been confronted with the health issue of snakebite and the lack of effective treatment in the rural areas of Brazil. He thereafter proceeded to study the venom of Brazilian snake species at the Bacteriological Institute of São Paulo (35). His work on venom extraction, yields, envenomation physiology, as well as immunization and immune serum production was published later in 1901. In his papers, Vital Brazil also demonstrated the specificity of the antiserum by showing that Calmette's anti-cobra serum was not effective against the venom of Brazilian *Bothrops* species, while antisera raised against venom of the Brazilian *Bothrops jararaca* as well as *Crotalus durissus terrificus* species proved to only be effective against that same respective venom that they had been raised against. By that time, Vital Brazil was producing anti-crotalic and anti-bothropic serum in mules and horses, as well as a mixture of the two, anti-ophidic serum, which was the first polyvalent antivenom. By setting up an exchange system with farmers—snakes in exchange for antivenom—Vital Brazil was able to distribute the serum in the rural areas around São Paulo and increase the general awareness of the public of the issue and treatment of snakebite, while getting new supplies of different snakes for venom extraction and research in exchange (37). With his pioneering work on antivenom and the crucial role he played in setting up two major institutes for research on snake venom and production of antiserum, the Butantan Institute in São Paulo and the Vital Brazil Institute in Rio de Janeiro, Vital Brazil is an important figure in the history of antivenom production.

While France and Brazil were the settings of the major breakthroughs in the beginning of antivenom development, soon, other countries (all with high numbers of snakebite incidences) started producing antivenom against native snakes. After first having been proposed but not realized by Hideyo Noguchi in 1909, antivenom production was introduced In the USA by Afrânio do Amaral from the Butantan Institute. The first antivenom against North American rattlesnakes was produced in 1927, which was followed by a polyvalent crotalid antivenom in 1953 and a coral snake antivenom in 1967. Australia started antivenom production against the tiger snake (*Notechis scutatus*) in 1930, and in the following 50 years, 11 new antivenoms against venomous snakes, fish, jellyfish, and spiders were introduced. Similarly, antivenom production was taken up in Costa Rica (where up until then, antivenoms produced in Brazil had been used) by the *Instituto Clodomiro Picado* in 1970, and South Africa, where a polyvalent antivenom against the puff adder (*Bitis arietans*) and Cape cobra (*Naja nivea*) was first introduced in 1932 (32, 42, 43). The first scorpion antivenom was developed in Mexico by Daniel Vergara Lope in 1906. However, it was not until 1926 that anti-scorpion serum was first produced for therapeutic use by Isauro Venzor and Carlos León de la Peña. The history of scorpion antivenoms is explored in detail by Boyer (44).

In addition to the introduction of new antivenoms, an important step forward was also enabled by various quality improvements. These include different antibody purification and enzyme digestion strategies to produce Fab and F(ab')_2_ fragments, such as CroFab® (a Fab-based antivenom against North American crotalid envenomings) and Anascorp® (a F(ab')_2_-based antivenom against envenomings caused by *Centruroides sculpturatus* venoms). The goal of these strategies was to minimize adverse reactions caused by the non-human nature of the antibodies, as well as refined immunization protocols (32, 45, 46). Furthermore, isolation techniques, biochemical and functional characterization of several venoms (47), as well as venomics have been instrumental in establishing a better scientific understanding of toxicity and venom-induced pathology. Although venomics was first described in 2004 as a venom proteomics methodology (48), the more recent definition encompasses the global study of the venom and the venom gland, incorporating characterization of the whole venom profile through integration of proteomic, transcriptomic, and genomic methodologies (49, 50). These techniques now allow researchers to more rationally design antivenoms and undertake research in the development of next-generation antivenoms (51).

Despite the cost-effectiveness of traditional antivenom manufacturing processes and considerable progress towards improving antivenom quality, the past and current shortage of commercial antivenom supplies, also known as the antivenom crisis, remains an unresolved issue (52). In 2014, the discontinuation of production of Fav-Afrique® (Sanofi Pasteur), a polyvalent antivenom previously used to treat envenomings caused by medically relevant snake species of sub-Saharan Africa, clearly exemplified the general lack of interest that the pharmaceutical industry has shown for products with low profitability (53). Many of the factors contributing to preserve this long-standing problem are directly or indirectly related to economic aspects, such as deficient antivenom distribution, insufficient research funding, and omission to appear on the agenda of public health institutions and policy makers (52, 53). Hopefully, the multi-component global strategy recently launched by the World Health Organization's (WHO) snakebite envenoming working group (SBE-WG), which aims at reducing morbidity and mortality caused by snakebite envenoming in the coming decade, will help drive the necessary actions to secure access and affordability of high-quality antivenoms in regions where it is urgently needed (54).

## The History of Antibody-Based Therapies

From the 1950s to the present, numerous breakthroughs in the fields of molecular biology, biochemistry, and immunology have laid the foundation for the development of antibody-based therapies. Back in 1953, James Watson and Francis Crick solved the molecular structure of DNA based on X-ray diffraction data and stereochemistry (55). A few years later, in 1958, the central dogma of molecular biology was postulated; the first correct proposal on how the transfer of information occurred between nucleic acids and proteins (56). Successive work along the same line resulted in the establishment of the genetic code and a description of how gene expression and protein translation occur (57–59). In the late 1960s, the discovery of bacterial restriction endonucleases (60, 61) opened up for the possibility of inserting foreign DNA to construct chimeric nucleic acids (62), initiating the revolution of recombinant DNA technology. Concomitantly, the first analysis of DNA nucleotide sequences were reported in 1972 (63–65), and since then, advancements in this field have boosted the progress of molecular biology and other related fields.

Following the discovery of antibody molecules and the elucidation of their structure (see section Important discoveries within antibody generation and characterization), in 1975, Köhler and Milstein reported the development of the hybridoma technology. Hybridomas are cultured cells that secrete monoclonal antibodies of predefined specificity, generated by fusing mouse myeloma and mouse B cells from an immunized donor mouse. This technique made it possible to isolate hybrid cell lines secreting different monoclonal antibodies targeting the same antigen. In addition, it enabled the obtainment of monoclonal antibodies of virtually any specificity after immunization of an animal (66). Due to the murine origin of monoclonal antibodies produced by this approach, subsequent research efforts were oriented towards the production of antibodies with a higher degree of homology to human antibodies. Functional chimeric antibodies were first obtained in 1984 by designing immunoglobulin genes consisting of mouse variable regions and human constant regions using recombinant DNA technology. The inclusion of human constant regions was intended for better effector functions and less immunogenicity (67, 68).

The next major breakthrough in the history of antibody discovery took place in 1985 with the creation of the filamentous fusion phage. Phages displaying foreign peptides on their surface were constructed by fusion of the phage gene III DNA sequence with a foreign DNA fragment. Isolation of specific filamentous phages based on binding affinity of the foreign peptide towards an antibody, when available, established this strategy as a convenient method for isolating and amplifying a gene from a library of random fragments. Additionally, since fusion phages retain infectivity and immunogenicity, this also became a suitable approach for raising antibodies against foreign peptides (69). Shortly after (1986), bacterial surface display emerged as an alternative display technology (70).

Another invention with great impact for DNA cloning was the PCR, also reported in 1986. This reaction allowed *in vitro* amplification of specific DNA segments through repetitive cycles of denaturation, hybridization, and polymerase-mediated extension (71). PCR has now become an essential method for multiple applications, such as sequencing, diagnosis, and gene mutagenesis, to name a few (72). Continuing with the same rationale behind the transition from mouse antibodies to chimeric antibodies, the subsequent imminent step was to design more human-like antibodies, or so-called humanized antibodies. Humanized antibodies were first created in 1986 by replacing the CDRs in a human antibody scaffold with the CDRs of a mouse antibody (73). Humanized antibodies were expected to be less immunogenic than chimeric and fully heterologous antibodies due to their higher proportion of human protein sequence.

In search for novel *in vitro* selection methods for antibody discovery, in 1990, antibody phage display technology was developed as an application of the filamentous fusion phage (74). In this case, the genetic sequences of the immunoglobulin variable domains (single-chain variable fragments, scFvs) were fused with the DNA sequence of the phage gene III, allowing the variable domains to be expressed on the surface of fd bacteriophages, thereby conveniently linking genotype with phenotype through the phage. When an antigen of interest is available, antibody phage display technology enables the selection and isolation of high affinity scFv binders (74). Since its development and until now, the construction of large naïve, immune, synthetic, and semi-synthetic libraries as a source of antibodies, of animal or human origin, has positioned antibody phage display technology as an extremely powerful tool for high-throughput antibody discovery (75). To date, more than 10 monoclonal antibodies derived from phage display experiments have successfully entered the market for a diverse range of therapeutic indications. Of these, it is worth highlighting the first approved fully human antibody (adalimumab), which is an anti-TNF-α antibody for treating rheumatoid arthritis and other inflammatory diseases and remains the top selling pharmaceutical of the global market (76).

In 1993, Hamers-Casterman et al. reported the discovery of heavy-chain only antibodies (HCAbs), which are naturally present in camelids (i.e., camels, llamas, dromedaries, and alpacas) (77). Later on, further research confirmed that the variable domain of these antibodies (V_H_H) alone, with a size of about 12–15 kDa, is still functional, turning it into the smallest antibody fragment with antigen binding capacity (78). Notably, these single-domain antibodies (also known as nanobodies) possess important structural differences in the antigen binding region compared to conventional antibodies, which may provide them with an ability to recognize cryptic (hidden) epitopes (79). Due to their specific antigen binding capacity, together with their high solubility, stability, and ability to penetrate rapidly into deep tissue, among other favorable characteristics (78), several therapeutic candidates based on single-domain antibodies have recently been under clinical investigation. Of note, last year, caplacizumab, an anti-von Willebrand factor, became the first nanobody-derived therapy to gain regulatory approval, and it is now on the market (80). HCAbs were also discovered in 1995 in cartilaginous fish (i.e., sharks, rays, skates, and chimeras). From these antibodies, variable new antigen receptor (VNAR) domains similar to V_H_Hs in terms of structure and function were described (81). Although less studied than V_H_Hs, VNARs also hold potential for therapeutic applications (82).

Transgenic mice carrying human immunoglobulin loci were developed in 1994 as an innovative approach for obtaining human antibodies using hybridoma technology (83–85). This method allows for the generation of fully human monoclonal antibodies with the advantage of maintaining the processes of natural recombination and affinity maturation that occur *in vivo* (86). The XenoMouse® is a commercial example of such approach (87), and the first from which a pharmaceutical on the market has been derived, namely panitumumab, a human monoclonal antibody against epidermal growth factor receptors (88). Along with the different display techniques, the use of transgenic mice circumvents many of the drawbacks of therapeutic murine, chimeric, and humanized antibodies, such as suboptimal pharmacokinetics and unfavorable immunogenicity, mainly due to the fully or partial heterologous nature of the latter mentioned antibody formats. Other transgenic animals have also been developed for the same purpose, including rats, rabbits, and calves (89, 90). It is also worth mentioning that in the late 1990s, other display technologies, different from phage and bacterial display, were developed, such as yeast surface display (91), ribosome display (92), and mRNA display (93). Within these types of technologies, CIS-display and mammalian cell surface display appeared last, in 2004 (94) and 2006 (95), respectively. These new display technologies were each invented to either circumvent issues of amplification biases or better allow for selection of “druggable” antibodies early in the discovery process.

Nowadays, the current molecular biology tools together with the above-mentioned biotechnological progress have made it possible to design and express antibody-based proteins in a vast repertoire of molecular formats. Conventional formats already in use as antitoxins, either experimentally or in the clinic, include whole IgG, F(ab')_2_, Fab, diabody, scFv, and V_H_H (96). Besides these formats, in fields different from envenoming therapy, antibody engineering has led to diverse multivalent and multispecific constructs (97, 98). For example, ALX-0171 is a trimeric nanobody against respiratory syncytial virus currently under development (99). Also, bispecific antibodies showing neutralizing capacity *in vitro* and *in vivo* against filoviruses have been reported (100). Additionally, other binding proteins may be investigated for their potential to neutralize toxins (101). Favorably, the field of antivenom research now has an opportunity to build on top of decades of progress on design and engineering of biotherapeutic agents to generate high affinity toxin-neutralizing molecules with unprecedented neutralizing capacity, low immunogenicity, and desirable pharmacokinetics (102, 103).

## Advances in Antivenom Research

Despite the advancements that have occurred in the field of biotechnology since Calmette's first steps towards the introduction of antiserum therapy as a treatment for animal envenoming, to this date, antiserum remains the only effective treatment against envenomings caused by venomous animals (2). Many barriers are likely to have contributed to this, including the neglected character of the problem, the complexity of developing an alternative treatment, and the low economic incentive for companies to develop treatments against envenomings. However, in 2017 snakebite envenoming was recognized by its official addition to the list of Category A Neglected Tropical Diseases by the WHO (104). This may possibly help create the necessary awareness, political will, and incentives to help researchers develop novel therapies against snakebite and other envenomings (54, 102). Nevertheless, over the last many years, academic research groups across the world have been attempting to use the last decades of biotechnological advancements to improve current or develop novel treatments against animal envenomings. Many of the avenues that have been, or still are being pursued toward the development of alternative therapies to current antivenom treatment, include many different types of monoclonal antibodies (96) and several types of non-antibody-based molecules, such as oligonucleotide aptamers (105, 106), nanoparticles (107), peptides (108), naturally occurring protein inhibitors (109–116), and small molecule inhibitors (117–119). Varespladib and batimastat are examples of small molecule inhibitors originally developed against indications outside the field of snakebite envenoming that were later shown to inhibit toxic effects from phospholipases A_2_ and snake venom metalloproteinases, respectively (117, 119, 120). Both peptides, naturally occurring non-antibody proteins, and nanoparticles have also been shown to have neutralizing capacities against snake venoms (121). However, none of these molecules have ever reached the clinic, and they fall outside of the scope of this review.

Within the scope of this review, the development of novel envenoming therapies based on monoclonal antibodies is being pursued using many of the technologies presented in previous sections of this review. One technology that has been employed numerous times to discover monoclonal antibodies against animal toxins is hybridoma technology (1). In the field of envenoming, it was first used in 1988 by Bahraoui et al. to discover monoclonal antibodies against toxin II from the scorpion *Androctonus australis Hector*. In this study, mice were immunized with toxin II, spleen cells were fused with myeloma cells, and the resulting hybridomas were tested for secretion of toxin-binding monoclonal antibodies. The obtained antibodies were tested for their ability to prevent lethality after lethal amounts of toxin were preincubated with each antibody and injected intracerebroventricularly in mice. One monoclonal antibody neutralized toxin doses as high as 50 LD_50_s (122). Other groups have since then employed murine hybridomas for discovery of monoclonal antibodies against many other animal toxins (123, 124). Similar to the transition from the use of monoclonal antibodies of animal origin to human origin in other antibody research fields, human monoclonal antibodies have also gained increasing interest within antivenom research. To the best of our knowledge, to this date, only one example of the discovery of human IgGs against snake toxins using transgenic mice has been reported. In 2008, transgenic mice were used to discover human IgGs against the metalloproteinase HR1a from *Protobothrops flavoviridis* (a pit viper from Ryukyu Islands of Japan) by Morine et al. In that study, 300 hybridoma cell fusions were screened for production of toxin-binding IgGs, and of these, 80 antibodies were identified as HR1a-reactive. The IgGs were tested for their ability to inhibit proteolytic and hemorrhagic activity *in vitro*, where some showed the ability to partially inhibit both toxic effects (125).

Another technology that has been utilized by different research groups to discover monoclonal antibodies of different origin against toxins from snake (126), spider (127), scorpion (128), and bee (129) venoms is antibody phage display. Antibody phage display technology was first used for the discovery of monoclonal antibody fragments against animal toxins in 1995 by Meng et al. (126). The authors used an scFv library generated from spleen cells of mice that had been immunized with crotoxin obtained from the snake *Crotalus durissus terrificus*. The affinity matured library was used to discover scFvs with specificity to crotoxin. The scFvs were tested in lethality assays in mice upon preincubation with lethal doses of Mojave toxin, demonstrating the ability of the scFvs to provide prolonged survival in mice. Since the first use of antibody phage display technology in toxinology, this discovery methodology has been employed by several groups within the field, and some groups have reported the discovery of V_H_H monoclonal antibody fragments from phage display libraries generated from both non-immunized and immunized llamas against animal toxins (130, 131).

The first report on the use of antibody phage display technology for generating a human monoclonal antibody fragment against a snake toxin was made by Lafaye et al. in 1997 (132). Here, scFvs from a human semi-synthetic antibody phage display library were discovered against crotoxin, and these antibodies were demonstrated to bind the toxin in ELISA experiments. Eight years later, in 2005, the first report on the discovery of a human monoclonal antibody fragment against a scorpion toxin using phage display technology was published by Riaño-Umbarila et al. (133). In this study, human scFvs were discovered against Cn2 from *Centruroides noxius*, using a library constructed from a single naïve human donor. Following four rounds of selection, an scFv was affinity matured using directed evolution. In a subsequent lethality assay, where the most promising affinity matured scFvs were incubated with either toxin or whole venom prior to intravenous injection into mice, one scFv demonstrated the ability to prevent lethality of 2 LD_50_s of venom and toxin. In 2011, Funayama et al. published the first paper on discovery of human scFvs against bee venom toxins using phage display technology (129). In their study, a naïve human scFv library was used to select monoclonal antibody fragments against melittin and phospholipase A_2_ from Africanized honey bees. A combination of two of the resulting scFvs was reported to inhibit myotoxic effects *in vivo* and prolonged survival of mice in lethality assays, where venom and scFvs were preincubated prior to administration. Since these first discoveries of human monoclonal antibody fragments against animal toxins, phage display technology has been used to discover human monoclonal antibody fragments against toxins from other snakes (134–136), scorpions (128, 137), and bees (129, 138). To the best of our knowledge, no human monoclonal antibody fragment has yet been discovered against a spider toxin using phage display selection. In 2018, Laustsen et al. reported the first development of fully human monoclonal IgGs against animal toxins using antibody phage display technology. The IgGs were discovered from a naïve human library of scFvs and had specificity to dendrotoxins from *Dendroaspis polylepis* (the black mamba). The monoclonal IgG antibodies were demonstrated to individually provide full protection (100% survival) in rodents when the antibodies were co-administered intracerebroventricularly upon preincubation with lethal doses of venom fractions. Moreover, with their study, Laustsen et al. were also first to explore the use of oligoclonal IgGs against animal toxins by demonstrating that two different mixtures of three and four IgGs could fully prevent lethality of whole venom when mice where challenged by the intracerebroventricular route with lethal doses of venom preincubated with the oligoclonal antibody cocktails (139).

Using phage display technology, research groups have attempted to take antibody discovery a step further by engineering monoclonal antibody fragments to be specific to more than one toxin. This phenomenon is referred to as antibody cross-reactivity, which in relation to animal toxin neutralization is a desirable antibody property, as animal venoms are complex mixtures of toxins of both high and low homology (140). Being able to use only one monoclonal antibody to target two or more toxins will help lower the total number of monoclonal antibodies needed for a recombinant antivenom based on oligoclonal antibodies, which in turn will improve developability and cost of manufacture (102, 103, 141–143). In this regard, Pucca et al. demonstrated that a unique human monoclonal antibody fragment could neutralize α and β-toxins (Ts1, Ts2, Ts5, CssII, and LqhIII) from different scorpion genera (*Tityus serrulatus, Centruroides suffusus suffuses*, and *Leiurus quinquestriatus hebraeus*) (137). Similarly, in the work of Roncolato et al., human scFvs discovered against *Bothrops jararacussu* venom toxins were shown to cross-neutralize phospholipases A_2_ from the venoms of other species (*Bothrops jararaca, Bothrops neuwiedi*, and *Bothrops moojeni*) (144). Silva et al. have also demonstrated the ability of human scFvs to cross-neutralize the toxic effects of bothropic and crotalic venoms (136). Using a different approach based on semi-rational design, recently, Riaño-Umbarila et al. mutated the gene encoding a human scFv targeting Cn2 from *C. noxius* scorpion venom. The gene was mutated in selected residues of the CDR3 region, where upon a new antibody library was constructed and used to select binders against other toxins from *C. noxius*, as well as other scorpions. A resulting scFv displayed neutralizing abilities against 13 neurotoxins present in the venoms of nine different species of Mexican scorpions (145).

In combination, the many reports on the discovery of a different types of monoclonal antibodies against a multitude of different toxins from venomous animals demonstrate that increased interest and application of newer biotechnological approaches and techniques are building in the field of envenoming therapy research. Although many of these developments are yet to enter the clinical setting, the future perspectives for this field are improving. It should, however, be noted that recombinant antivenoms based on oligoclonal antibodies may possess somewhat different therapeutic properties than traditional antivenoms based on polyclonal heterologous antibodies. Oligoclonal antivenoms are much simpler in composition, making them less likely to exhibit cross-neutralization properties to the extent of having paraspecificity (cross-reactivity to venoms that were not part of the development or manufacturing process for an antivenom). It is therefore essential that recombinant antivenoms are designed to include monoclonal antibodies that can neutralize all the medically relevant toxins in a given whole venom and/or abrogate toxin synergism for such venom (146–148). As many medically relevant venomous animals possess up to dozens of medically important toxins, the possibility of engineering the cross-reactivity of monoclonal antibodies, as well as oligoclonal mixtures thereof, may be key to successful recombinant antivenom design (140, 149).

## Future Perspectives

With the renewed focus on snakebite envenoming as a Category A Neglected Tropical Disease by the WHO, there is a hope that the development of much needed therapies against both snakebite and other animal envenomings will become increasingly incentivized for researchers worldwide. Among the scientific and technological fields that are expected to gain increased interest, the development of standardized approaches for rational engineering of cross-reactivity for both individual monoclonal antibodies and oligoclonal antibody mixtures is likely to gain traction, as this is an essential parameter for creating broadly-neutralizing recombinant antivenoms that can be used against multiple species (140). Also, manufacturing strategies for low cost production of recombinant antivenoms will need to be further developed. Moreover, the field of antivenom development has only recently seen the introduction of systematic and holistic strategies for developing recombinant antivenoms (51, 102, 141, 142), and these strategies need to be both strengthened and further tested in the laboratory setting. Finally, the entire field of envenoming diagnostics has seen very little innovation for decades, and an opportunity exists for implementing both bio and nanotechnologies for the development of novel diagnostic tools and devices that can help stratify envenoming cases and quantitatively monitor pathogenesis of envenoming (54).

## Author Contributions

FC, RJ, EB-M, and LL wrote part of the review and provided critical feedback. FC was in charge of drawing the figures. MP and AL designed the review, wrote part of the manuscript, and provided revisions. JB gave his valuable and professional suggestions and revised the manuscript. All authors read and approved the final manuscript.

### Conflict of Interest Statement

The authors declare that the research was conducted in the absence of any commercial or financial relationships that could be construed as a potential conflict of interest.
